# Rapid Determination of *β*-Glucan Content of Hulled and Naked Oats Using near Infrared Spectroscopy Combined with Chemometrics

**DOI:** 10.3390/foods11010043

**Published:** 2021-12-24

**Authors:** Maninder Meenu, Yaqian Zhang, Uma Kamboj, Shifeng Zhao, Lixia Cao, Ping He, Baojun Xu

**Affiliations:** 1 Food Science and Technology Program, BNU-HKBU United International College, Zhuhai 519087, China; meenu_maninder@yahoo.com (M.M.); yaqianzhang_007@link.cuhk.edu.hk (Y.Z.); 2 School of Chemical Engineering and Physical Sciences, Lovely Professional University, Jalandhar 144411, India; amu_kam@yahoo.com; 3 Zhangjiakou Academy of Agricultural Sciences, Zhangjiakou 075000, China; zsf1886@163.com (S.Z.); ymsclx@163.com (L.C.); 4 Statistical Program, BNU-HKBU United International College, Zhuhai 519087, China; heping@uic.edu.cn

**Keywords:** NIR spectroscopy, *β*-glucan, oats, prediction, partial least square regression

## Abstract

The quantification of *β*-glucan in oats is of immense importance for plant breeders and food scientists to develop plant varieties and food products with a high quantity of *β*-glucan. However, the chemical analysis of *β*-glucan is time consuming, destructive, and laborious. In this study, near-infrared (NIR) spectroscopy in conjunction with Chemometrics was employed for rapid and non-destructive prediction of *β*-glucan content in oats. The interval Partial Least Square (iPLS) along with correlation matrix plots were employed to analyze the NIR spectrum from 700–1300 nm, 1300–1900 nm, and 1900–2500 nm for the selection of important wavelengths for the prediction of *β*-glucan. The NIR spectral data were pre-treated using Savitzky Golay smoothening and normalization before employing partial least square regression (PLSR) analysis. The PLSR models were established based on the selection of wavelengths from PLS loading plots that present a high correlation with *β*-glucan content. It was observed that wavelength region 700–1300 nm is sufficient for the satisfactory prediction of *β*-glucan of hulled and naked oats with R^2^c of 0.789 and 0.677, respectively, and RMSE < 0.229.

## 1. Introduction

Oats are produced in various countries around the world. According to the Food and Agriculture Organisation of United Nations, Russia is the top producer of oats (4.72 million metric tons (mmt)) followed by Canada (3.44 mmt), Spain (1.48 mmt), Australia (1.23 mmt), Poland (1.17 mmt), and China (1.00 mmt) [1]. There are about seventy species of oats around the world, while hulled oats (*Avena sativa* L.) and naked oats (*A. nuda* L.) are the most cultivated oats species. The latter is hull-free and exhibits a higher energy level compared with hulled oats [2]. Oats are a rich source of soluble fibers, mainly, *β*-glucan [3]. Primarily, *β*-glucan is present in the cell walls of the aleurone layer and starchy endosperm of oat grains [4]. It is a homopolysaccharide of long linear chains of glucose units connected by *β*-(1→3) and *β*-(1→4) linkages [5,6].

*β*-glucan is also known as a functional and bioactive food ingredient due to its potential health benefits such as antitumor, anti-inflammatory, antioxidant, hypoglycemic, hypocholesterolemic, and immunomodulatory activities [3,5]. Thus, the demand for food products with a high level of *β*-glucan has been increased worldwide. Oats are used as human food in the form of cereals, breads, beverages, and infant foods [3]. *β*-glucans are also employed in the pharmaceutical industry to produce wound dressing material; for the treatment of burn injuries, skin substitute, and bone-substituting material; and in arthritis treatment. Moreover, *β*-glucans are also used in the cosmetics industry due to their anti-aging, moisturizing, and wound healing effects [5,7]. However, high *β*-glucan content is undesirable in the case of monogastric animal feed due to the digestion problem and reduced nutritional value of the feed [8].

To develop a new plant variety with a high level of *β*-glucan, the quantification of *β*-glucan is also important for plant breeders for the selection of high-quality oat cultivar. The McCleary enzymatic method is the most widely used method for the determination of *β*-glucan [9]. The primary step of this method involves disintegration and homogenization of oat grains that may induce variations in the *β*-glucan measurement if they are not properly conducted. Moreover, this chemical method is destructive, requires a long determination time, and is costly. During plant breeding experiments, extensive chemical analysis has to be conducted to explore the target genotype. Thus, there is an immediate requirement to substitute conventional analysis with up-to-date non-destructive, chemical-free, rapid, and easy techniques.

Near-infrared spectroscopy (NIRS) meets the requirements of plant breeders and food scientists to detect the *β*-glucan of oats without chemicals, in less time, and with minimum sample preparation and cost. NIR spectrum ranges from 700 to 2500 nm consist of broad overtone and combination bands of certain fundamental vibrations of C–H, N–H, and O–H bonds present in organic compounds of grain samples [10]. NIRS in conjunction with chemometrics has been successfully employed in medical science [11], pharmaceuticals [12], and food industries [10,13,14]. In recent decades, NIRS along with modified partial least square regression was used for the accurate prediction (coefficient of determination for calibration (R^2^_C_) of 0.80 and coefficient of determination for prediction (R^2^p) of 0.75) of groat percentage in oats from Italy [13]; neutral detergent fiber, acid detergent fiber, and acid detergent lignin of oats collected from Italy (R^2^_C_ = 0.92–0.95 and coefficient of determination for cross-validation (R^2^cv) = 0.83–0.93) [14]; nitrogen (R^2^c = 0.973, coefficient of determination for validation (R^2^v) = 0.984) and protein content (R^2^c = 0.973, 0.965, R^2^v = 0.984, 0.973) of food products containing oats [15], protein, and lipid contents of oats from Brazil [16]; and the detection of oat flour adulteration with wheat flour [17]. Previously, NIRS in the region 1194–1240 nm was also successfully employed to predict the accumulation of (1→3) (1→4)-*β*-D-glucan while grain filling in barley [18]. In addition, NIRS was also employed to predict the *β*-glucan content of different oats genotypes collected from Italy and other European countries [4,19]. Previously, different types of NIR Instruments were explored for their suitability in measuring *β*-glucan content in naked barley. The Fourier transform near-infrared (FT-NIR) reflection instrument was reported to predict *β*-glucan with high efficiency (R^2^c = 0.96–0.98) [20]. Furthermore, the NIR spectrum of 288 seeds of whole oat groats for six varieties grown in the USA was collected and used for the prediction of *β*-glucan content by employing Partial Least Square regression (PLSR) analysis [21]. Recently, NIR spectroscopy in conjunction with chemometrics was also employed for the efficient prediction of *β*-glucan content of hulled oats samples collected from diverse geographical regions of the USA [22].

However, to the best of our knowledge, no study has been conducted for the prediction of *β*-glucan content of hulled and naked oats collected from China. Thus, this study was carried out to predict the *β*-glucan content of oats from China, by using NIRS in conjunction with PLSR analysis. In this study, we also included certain oat samples collected from the United States and Canada to increase the genetic and geographical variability of the sample pool.

## 2. Materials and Methods

### 2.1. Collection of Oat Samples

A total of 179 different varieties of oat samples (100 naked and 79 hulled oats) were procured from Zhangjiakou Agricultural Academy, Hebei Province, China. The samples were cleaned manually to remove unwanted materials and damaged grains and then refrigerated at (2–6 °C) until further analysis. The samples were brought at room temperature before chemical and spectral analysis followed by dehulling and removal of impurities. The final oat groats were finely ground by a regular laboratory mill (400A, Yongkang City Zhanfan Industry and Trade Co., Ltd., Yongkang, Zhejiang, China) and passed through a 60 mesh screen (d = 0.25 mm) to achieve homogeneous particle size that, in turn, reduce the problem of scattering during NIR data acquisition. The resultant oat flours were further used for chemical and spectral analysis.

### 2.2. Chemicals

The analytical grade sodium dihydrogen orthophosphate dihydrate (NaH_2_PO_4_^.^2H_2_O), sodium hydroxide (NaOH), and glacial acetic acid were purchased from Tianjin Damao Chemical Reagent Co., Ltd. (Tianjin, China). The *β*-glucan content of oat samples (reference data) was determined by using the mixed-linkage beta-glucan kit (K-BGLU, Megazyme, Bray, Ireland).

### 2.3. Determination of β-Glucan Content

The mixed-linkage *β*-glucan assay procedure (McCleary method) was used to determine the *β*-glucan content of oat samples [9]. Briefly, the 120 mg of oat sample was suspended in 0.2 mL of 50% ethanol and hydrated in a sodium phosphate-buffered solution (4 mL, 20 mM, pH 6.5). Furthermore, the reaction mixture was incubated with purified lichenase enzyme (0.2 mL) and filtered. An aliquot of the filtrate was then hydrolyzed with purified *β*-glucosidase (0.1 mL in 50 mM sodium acetate buffer pH 4.0) to release D-glucose followed by the addition of glucose oxidase/peroxidase reagent (3.0 mL) and incubation for 20 min at 50 °C. The absorbance of the solution was measured at 510 nm within 1 h. The *β*-glucan content of each sample was analyzed in triplicates.

### 2.4. Acquisition of NIR Spectra

The NIR spectrum of the oat flour sample was measured by using Frontier FT-IR/NIR Spectrometer (PerkinElmer Inc., Waltham, MA, USA) using a NIR infrared reflectance accessory—NIRA (PerkinElmer Inc., Waltham, MA, USA) equipped with a spinning sample module and Spectrum-V.7.3.1.1023 (PerkinElmer Inc., Waltham, MA, USA) software. The spectrum was recorded in diffuse reflectance mode from 700 to 2500 nm using 32 scans at 1 nm intervals. The measurements were performed in duplicate, and the average spectrum was used for further analysis.

### 2.5. Statistical Analysis

The interaction of NIR radiation with oat samples gives rise to a complex spectrum. This spectrum comprises overtone vibrations and combination bands at particular wavelengths due to the bonds present in organic compounds of oats. Thus, for the extraction of useful information from spectra and quantitative analysis of *β*-glucan present in samples, multivariate statistical analysis was carried out by employing reference data of *β*-glucan as primary data and spectral data as secondary data. The multivariate analysis was performed by using chemometric software (The Unscrambler X version 10.3; CAMO Software AS, Oslo, Norway). Random sampling was conducted to divide oats samples into two categories—calibration and prediction set with the ratio of 4:1. In case of hulled oats, 64 samples were included in the calibration set, whereas 15 samples were kept in the prediction set. However, in the case of naked oats, 80 samples were kept in the calibration set and 20 samples were used to develop the prediction model [10]. The spectral data were maximum normalized, and a baseline correction was performed for the obtained spectra. Savitzky–Golay smoothening was applied to the spectral data. The complete NIR wavelength range was divided into three equal parts and equal width (for both naked and hulled oat samples) namely, 700–1300 nm (I), 1300–1900 nm (II), and 1900–2500 nm (III) followed by developing a correlation matrix for each section of wavelength with the *β*-glucan data of oat samples obtained by using reference analysis. PLSR and interval PLS regression (iPLS) was applied using Nonlinear Iterative Partial Least Squares (NIPALS) algorithm, and PCA analysis was performed on the spectral data as a whole and on the three parts each. Regression models were developed for each part for the determination of the *β*-glucan content in the samples.

## 3. Results

The McCleary method was used as a reference method for the determination of *β*-glucan content in seventy-nine oats varieties. The *β*-glucan content and moisture content of hulled and naked oat varieties under investigation are mentioned in Table 1 and Table 2. The moisture content of hulled and naked oat samples varied from 4.86% to 6.43%, and 4.17% to 6.19%. In the case of hulled oats, the highest amount of *β*-glucan (5.5%) was observed in ZNY 258 followed by 5.25% in the case of zyp2 gp 63-56, and 4.94% in the case of zyp2 gp 86-79, whereas oat variety Zhangyan #7 presented the lowest amount of *β*-glucan (3.1%). In the case of naked oats, the highest amount of *β*-glucan (5.22%) was found in sample BY 4, followed by 5.21% in Bayan #9 and by 5.17% in BY 34. However, the lowest content (3.12%) was observed in the case of sample BY 37.

**Figure 1:**
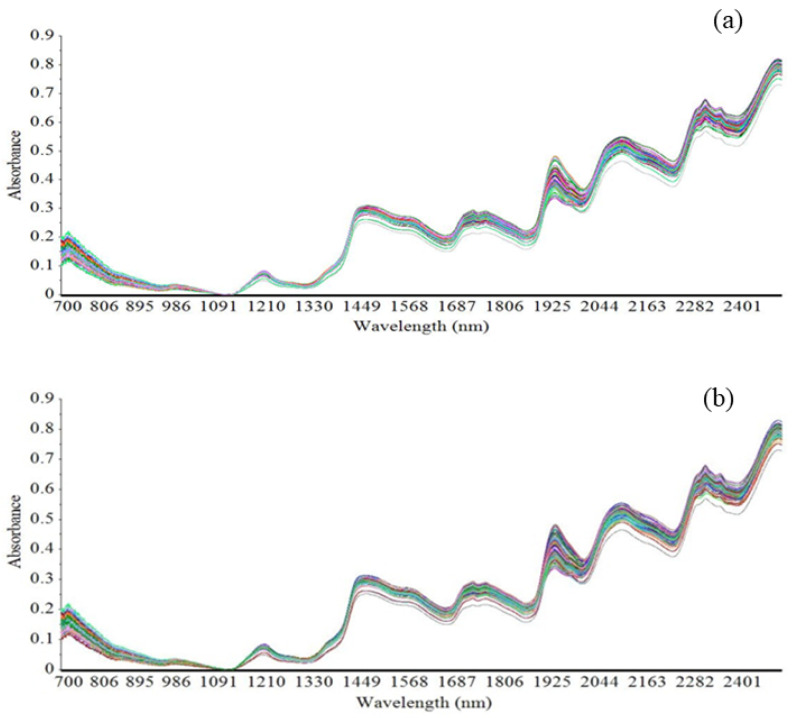
Typical near-infrared spectra of (**a**) hulled oat samples and (**b**) naked oat samples.

Figure 1 presents the NIR spectra of the hulled and naked oat samples. The absorption bands of both hulled and naked oat samples exhibit the least difference. The NIR spectra of samples were complex and present the prominent absorption bands around 1200 nm, 1450 nm, 1750 nm, 1950 nm, 2100 nm, 2300 nm, and 2500 nm, whereas the lowest absorbance was observed in wavelength band 900–1091 nm.

**Figure 2:**
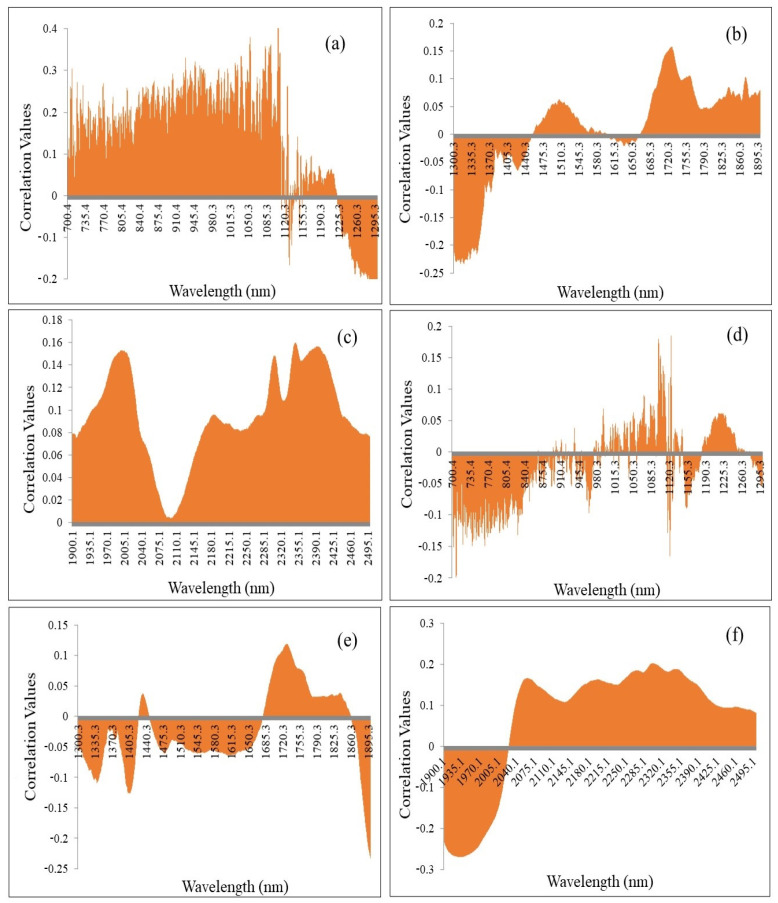
The correlation matrix plots of *β*-glucan content and NIR spectra in the wavelength ranges (**a**) 700–1300 nm, (**b**) 1300–1900 nm, and (**c**) 1900–2500 nm of hulled oats and (**d**) 700–1300 nm, (**e**) 1300–1900 nm, and (**f**) 1900–2500 nm of naked oats.

Thus, iPLS was performed for the selection of important wavelengths for the prediction of *β*-glucan in oats. Furthermore, the correlation matrix plots were employed to determine the correlation between each wavelength range and the *β*-glucan content. The correlation between wavelength range 700–1300 nm and 1400 to 1700 nm with the *β*-glucan content of hulled oat samples are shown in Figure 2a or Figure 2b, respectively. It can be observed that the wavelengths ranging from 1091 to 1218 nm present low correlation values (less than 0.2) compared with the other wavelengths (equal to greater than 0.2). The wavelengths from 1400 to 1700 nm also exhibit negligible correlation with *β*-glucan measurements. Thus, these wavelengths with less correlation were least important and considered as outliers for the determination of *β*-glucan to reduce the error, whereas the wavelength range 1300–1400 nm presented a strong negative correlation and the wavelength range 1700–1900 nm exhibits a strong positive correlation with *β*-glucan content. Thus, these wavelength ranges in group II with strong correlation were important for the prediction of *β*-glucan. Similarly, the important wavelengths (1950–2050 and 2150–2500 nm) were identified from group III (1900–2500 nm) using the correlation matrix, as shown in Figure 2c. The correlation matrix plots between *β*-glucan and the different wavelength ranges for the naked samples are presented in Figure 2d–f. As shown in Figure 2e or Figure 2f, wavelength ranges 1850–1865 nm and 1900–2000 nm exhibit a negative correlation with *β*-glucan, respectively. The wavelengths with correlation values of more than 0.1 were used for the development of a model, and the other wavelengths with low correlation values were considered outliers for the development of regression model.

**Figure 3:**
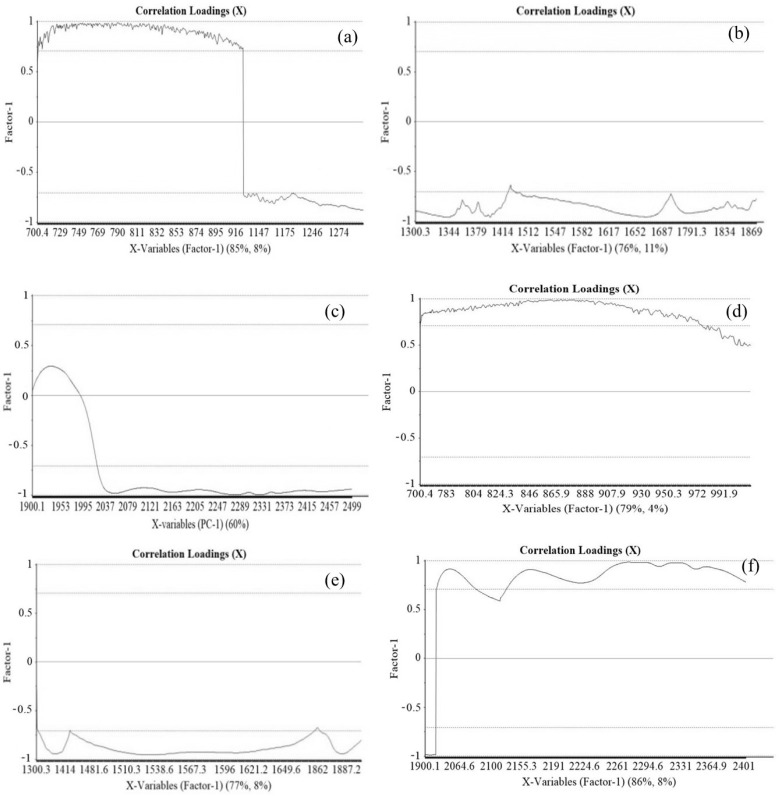
PLS loading plots in wavelength ranges (**a**) 700–1300 nm, (**b**) 1300–1900 nm, and (**c**) 1900–2500 nm for hulled oats and (**d**) 700–1300 nm, (**e**) 1300–1900 nm, and (**f**) 1900–2500 nm for naked oats.

The PLSR analysis was employed to determine the relationship between spectral absorbance and concentration of *β*-glucan by employing wavelengths extracted from iPLS. The Savitzky–Golay smoothening and normalization were applied to the spectral data before prediction analysis for reducing errors and for smoothening the spectral data. PLS loading plots for the hulled and naked samples are shown in Figure 3a–f, respectively. The loading plot shows the correlation loading coefficients for the regression equation developed to predict the *β*-glucan content in the oat samples. Each graph is divided into three parts: the upper part for the correlation loading value of 0.7–1 represents the wavelengths with a strong positive correlation coefficient with *β*-glucan content, the middle part shows wavelengths with moderate or small correlation coefficients, and the lower part shows strong negative correlation coefficients. Wavelengths with strong positive and negative correlations were selected for the prediction model, and the PLSR analysis was applied for the three groups separately.

The correlation loading plot for the naked samples (Figure 3d–f) showed that the wavelengths from 700–972 nm and 2100–2500 nm exhibit a strong positive correlation with the *β*-glucan of oats, whereas the wavelengths from 900–1300 nm present strong negative correlation coefficients with the *β*-glucan of naked oats. In the case of hulled oat samples, the correlation loading plots showed that the wavelength range from 700–900 nm exhibits a strong positive correlation and that the wavelength ranges 900–1900 and 2000–2500 nm present strong negative correlations (Figure 3a–c). Furthermore, it was also observed that the wavelength range 1900–2000 nm exhibits minimal to no correlation with *β*-glucan of hulled oat samples.

**Figure 4:**
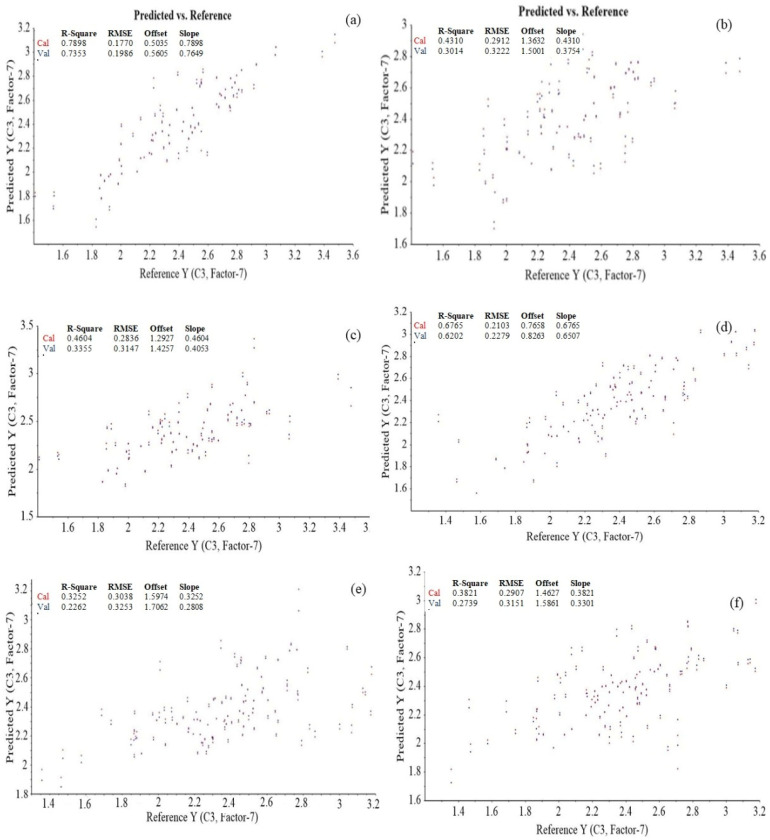
Partial least square model of *β*-glucan content in the wavelength ranges (**a**) 700–1300 nm, (**b**) 1300–1900 nm, and (**c**) 1900–2500 nm for hulled oats and (**d**) 700–1300 nm, (**e**) 1300–1900 nm, and (**f**) 1900–2500 nm for naked oats.

The prediction results for the *β*-glucan content of hulled and naked oats samples are shown in Figure 4 and Table 3. The R^2^c and R^2^p for hulled oats was higher, and the root mean square error (RMSE) was also considerably lower in the case of the first interval ranging from 700 to 1300 nm. A similar trend was also observed in the case of the naked samples. It was also observed that the R^2^c and R^2^p was slightly higher, and RMSE was comparatively lower in the case of hulled samples compared with naked samples in each wavelength interval. Overall, PLSR analysis in wavelength range 700–1300 nm can be successfully employed for the prediction of *β*-glucan content of hulled oats with R^2^c = 0.789, R^2^p = 0.735, RMSEC= 0.177, and RMSEP = 0.199, whereas in the case of naked oat samples, the best prediction results were also observed in the wavelength range 700–1300 nm with R^2^c = 0.677, R^2^p = 0.620, and RMSEC= 0.210, RMSEP = 0.228, but the R^2^c and R^2^p values were found to be poor in the second and third intervals ranging from 1300–1900 and 1900–2500 nm in both naked and hulled oats.

## 4. Discussion

The growing interest in functional foods accelerates research on plant materials rich in *β*-glucan. The health benefits of *β*-glucan such as antitumor, antioxidant, immunomodulatory, hypocholesterolemic, hypoglycemic, and anti-inflammatory activities are widely reported in the literature [3,5]. Thus, exploration of rich sources of *β*-glucan will lead to the development of novel plant material and food products with a high amount of *β*-glucan that, in turn, will provide various health benefits to consumers. In the present study, the *β*-glucan content of 100 different varieties of hulled oats and 79 varieties of naked oats were explored by employing the standard McCleary method. In case of hulled oat samples, the highest amount of *β*-glucan (5.5%) was observed ZNY 258 from Zhangjiakou, Hebei, China, and the lowest amount (3.1%) was found in the case of variety Zhangyan #7, also collected from Zhangjiakou, Hebei, China, whereas in the case of naked oat samples, the highest amount (5.22%) was observed in the case of variety BY 4 and the lowest amount (3.12%) was found in the case of sample BY 37. Both of these oat varieties were also collected from Zhangjiakou, Hebei, China. The *β*-glucan content of oat samples observed in the present study coincides well with the findings of previous studies [4,8]. In addition, the coefficient of variation among the *β*-glucan values of hulled and naked oat samples was found to be 10.67% and 10.79%. Thus, the chemometrics model established based on the spectral and this reference data will be able to predict the wide range of *β*-glucan in different oats varieties. A similar approach was also applied in a previous study while developing prediction models for phenolic compounds based on the NIR spectroscopy [10].

The NIR spectra of hulled and naked oats were collected from 700 to 2500 nm. The NIR spectra of oat samples were complex due to the presence of overtone vibrations and a combination of bands of C–H (aliphatic), C–H (aromatic), C–O (carboxyl), O–H (hydroxyl), and N–H (amine and amide) bonds present in organic compounds present in the oat samples [10,14,21]. The major absorption bands observed around 1200 nm correspond to the C–H 2nd overtone; 1450 nm corresponds to the O–H 1st overtone and the –CH_2_, –CH_3_, and –CH=CH– 1st overtone; 1750 nm corresponds to the C=O stretch 2nd overtone; 1950 nm corresponds to a combination of the stretching mode and the deformation mode of H2O; 2100 nm presents a combination of the O–H deformation mode and the C–O stretching mode; 2300 nm presents the –CH_3_ combination; and 2500 nm corresponds to a combination of C–H and C–C stretching [10,13,22]. The lowest absorbance was observed in the wavelength band 900–1091 nm. Similar absorption bands were also reported previously in the case of NIR spectra of oat groats [13]. Thus, due to the complex nature of the NIR spectra of oat samples, a multivariate statistical analysis needs to be conducted using chemical data as primary data and spectral data as secondary data for a better interpretation of the spectra, for the extraction of useful information, and for a quantitative analysis of the chemical components present in a plant material [23].

It was also mentioned that the wavelengths corresponding to low absorbance bands may be considered unwanted as these wavelengths contribute towards error during the establishment of a statistical model for the prediction of *β*-glucan in oats [22]. Thus, to extract important wavelengths for the prediction of *β*-glucan, iPLS was performed. In iPLS, the whole spectral data were subdivided into non-overlapping equal sections that undergo PLSR modelling to retrieve the most useful wavelength range (with high correlation coefficient and low RMSE) for the prediction of a particular biochemical parameter [24]. The resultant correlation matrix plots present the relationship between a particular biochemical parameter and absorption at different wavelengths. The wavelengths with less correlation values are mentioned as least important for the prediction of a biochemical parameter under investigation and can be rejected because they describe noise or other minor components [25]. In present study, the complete wavelength range (700–2500 nm) was divided into three equal parts (700–1300, 1300–1900 and 1900–2500 nm), and iPLS was performed. The resultant correlation matrix plots in Figure 2a revealed the shift in correlation values from high positive correlation to high negative correlation with an increase in the wavelength. The wavelength range from 700–1100 nm showed a positive but inconsistent correlation, 1120–1220 nm exhibited no or minimal correlation, and wavelength range 1225–1300 nm presented a high negative correlation with the *β*-glucan content of hulled oats. Thus, the wavelengths from 1120 to 1220 nm were not included in the development of the PLSR model for the prediction of *β*-glucan content. Furthermore, a negative correlation was observed between *β*-glucan content and wavelength range 1300–1440 nm (Figure 2b), whereas the highest wavelength ranges, 2040–2140 nm, showed a positive and feeble correlation (Figure 2c) and thus was not employed for the establishment of the PLSR models. However, the wavelength ranges 1950–2050 and 2150–2500 exhibited a satisfactory positive correlation with *β*-glucan content. Thus, they were employed for PLSR model development for the prediction of *β*-glucan content of hulled oats. In addition, the correlation matrix plots for different wavelength ranges (700–1300, 1300–1900, and 1900–2500 nm) and *β*-glucan contents of naked oats are presented in Figure 2d–f. The initial wavelengths till around 975 nm (Figure 2d) were negative, followed by an inconsistent and slightly positive correlation from 980 to 1150 nm and slightly positive correlation from around 1190 to 1260 nm followed by a negative correlation from 1290 to 1420 nm and 1442 to 1685 nm and a slightly positive correlation between *β*-glucan content and wavelength range 1686 to 1860 nm for naked oat samples (Figure 2e), whereas wavelength range 1900–2000 nm exhibits a negative correlation and wavelength range 2040 to 2500 nm exhibits a positive correlation with *β*-glucan of naked oats, as shown in Figure 2f. The wavelengths with correlation values more than and equal to 0.2 were employed for the establishment of a model, whereas other wavelengths with low correlation values were considered outliers for the development of the regression model. A similar approach was also employed for the selection of important wavelengths for the development of efficient calibration and prediction models for phenolic compounds of mung bean [10].

Furthermore, a PLSR analysis was performed to elucidate the relationship between *β*-glucan of oat samples and spectral absorbance using wavelengths extracted from iPLS. Before applying PLSR analysis, the spectral data were pretreated by employing Savitzky–Golay smoothening and normalization techniques to effectively remove scattering effects and noises such as tilt, baseline-drift, and reverse [26,27]. PLSR was applied to the data by the NIPALS algorithm and cross-validation technique [28]. In the case of 79 hulled samples, 63 were used to develop the model and 16 samples were employed for validation of the model. However, among the 100 naked samples, 80 samples were employed for calibration and 20 were used for validation of the model. The data was processed using seven principal components. It was observed that 94% of the complete data can be employed for the regression using two orthogonal principal components. Furthermore, the line loading plots of the PLS wavelength ranges (a) 700–1300 nm, (b) 1300–1900 nm, and (c) 1900–2500 nm for hulled and naked oats were also explored to support the finding of correlation matrix plots. The results of loading plots well supported the results of correlation matrix plots. In the PLS loading plots, the wavelengths with correlation values in the range of 0.7 to 1 were regarded as important for the model development and prediction of *β*-glucan content compared with the wavelength with values from −0.7 to 0 to +0.7. It was also observed that the initial wavelengths in the range of 700 to 1300 nm exhibited a positive correlation above or equal to 0.7 (Figure 3a), whereas the wavelength range 1300 to 1900 nm exhibited a negative correlation value (Figure 3b). In addition, the wavelengths in range 1900–2500 nm were also found to be least important for the prediction of *β*-glucan content, as their correlation values were below 0.7 to 1 (Figure 3c). In the case of naked oat samples, the wavelength range 700–900 nm was found to be important for the prediction of *β*-glucan content as their correlation value was between 0.7 to 1 (Figure 3d), whereas wavelengths from 1300 to 1900 nm exhibited negative correlation values below −0.5 and are thus regarded as outliers (Figure 3e) and the wavelength range 2000–2500 nm exhibited a variable but positive correlation between 0.7 to 1 (Figure 3f). Thus, it may also be considered important for the prediction of *β*-glucan content. Overall, in the case of Figure 3a–c, the wavelengths from 700–916 nm exhibited the highest positive correlation coefficient, whereas the wavelength region 1300–1900 nm (Figure 3) exhibited strong negative correlation coefficients. The wavelengths with high positive and negative correlations have equal importance in the prediction of *β*-glucan contents. The wavelength region 2000–2500 nm also presented a negative correlation coefficient; thus, wavelengths higher than 2100 nm are important for *β*-glucan prediction. In the case of both hulled and naked oat samples, the wavelengths less than 900 nm exhibited a strong positive correlation value. The wavelengths showing high correlation values for *β*-glucan content are more important for its prediction in the samples. Thus, the wavelengths showing a strong correlation with *β*-glucan from Figure 2 and Figure 3 were chosen for the final PLSR model development.

Table 3 and Figure 4 present the results of the prediction results of *β*-glucan from the PLSR models. In each wavelength interval, the wavelengths presenting a high correlation with *β*-glucan content were employed for establishing the PLSR model for the oat samples. In contrast, the wavelengths with minimal or no correlation were considered outliers and not employed for the establishment of calibration models. It can be observed from Table 3 that the PLS prediction model results are in accordance with the results obtained from correlation loading and correlation matrix results. The wavelength interval from 700 to 1300 nm exhibits relatively good R^2^ values for calibration and prediction compared with the models developed from 1300–2500 nm in both hulled and naked oats. The high values for R^2^c and R^2^p and lower values for RMSE present the good performance of the calibration and prediction model [29,30,31]. In wavelength range 1300–1900 nm, the R^2^c and R^2^p values were poor for both hulled (0.431 and 0.301) and naked oat samples (0.325 and 0.226). Similar results were also observed in wavelength range 1900–2500 nm with R^2^c and R^2^p of 0.460 and 0.336 for hulled and 0.382 and 0.274 for naked oats, respectively, whereas in the spectral range from 700 to 1300 nm, the R^2^c and R^2^p for naked oats were 0.677 and 0.620, and the highest values for R^2^c (0.789) and R^2^p (0.735) and the minimum values for RMSEC (0.177) and RMSEP (0.199) were observed for the hulled oat samples. Previously, it was mentioned that the calibration models with R^2^c between 0.66 and 0.81 present satisfactory predictions whereas prediction of chemical properties with R^2^c < 0.5 are reported as being unreliable [32,33,34]. A study by Seefeldt et al. also reported the best correlation of *β*-glucan of barley with the wavelengths in these regions [18]. Thus, in the future, the spectral region ranging from 700 to 1300 nm can be efficiently employed for the prediction of *β*-glucan of hulled oats to save time, whereas this wavelength range can also be employed for the satisfactory prediction of *β*-glucan of naked oats. In addition, a rapid and low-cost NIR spectrometer can also be designed by employing filters corresponding to 700–1300 nm for this particular application.

## 5. Conclusions

The rapid and non-destructive prediction of *β*-glucan content of hulled and naked oats was carried out by employing chemical data of the *β*-glucan content of oats (primary data) and NIR spectral data of oats (secondary data). The important wavelengths for the prediction of *β*-glucan of oats were selected using iPLS. The regression analysis was run on parts of the spectral data to check the range of spectra showing high coefficients of correlation with low error, chosen to build the best regression model for the prediction of the *β*-glucan content. The iPLS prediction and correlation matrix plots revealed the importance of wavelength range 700–1300 nm for the accurate prediction of *β*-glucan contents in hulled and naked oats samples. Various pre-processing techniques such as Savitzky–Golay smoothening and normalization were applied before prediction analysis. Applied pre-processing techniques helped in smoothening the data, which further helped in the analysis to understand the complex spectra. The results obtained from PLSR analysis indicate that the chemical data of *β*-glucan in hulled and naked oats along with the NIR spectra can be used for an accurate prediction of the *β*-glucan content with R^2^c of 0.789 and 0.677 for hulled and naked samples, respectively, in the wavelength range of 700–1300 nm with RMSEC and RMSEP less than 0.229.

## Figures and Tables

**Table 1:** *β*-glucan content, moisture content, and origin of hulled oat samples.

S. No.	Sample Name	Moisture Content (%)	*β*-Glucan % *w*/*w* (Dry wt. Basis)	Origin
1	zyp2 gp 016-14	5.91 ± 0.04	4.74 ± 0.06	Zhangjiakou, Hebei, China
2	zyp2 gp 002-1	5.65 ± 0.04	4.14 ± 0.04	Zhangjiakou, Hebei, China
3	zyp2 gp 035-30	5.64 ± 0.04	4.65 ± 0.12	Zhangjiakou, Hebei, China
4	zyp2 gp 129-119	5.68 ± 0.04	4.35 ± 0.19	Zhangjiakou, Hebei, China
5	zyp2 gp 135-65	6.41 ± 0.11	4.77 ± 0.10	Zhangjiakou, Hebei, China
6	zyp2 gp -74	5.99 ± 0.67	4.63 ± 0.03	Zhangjiakou, Hebei, China
7	zyp2 gp 023-20	6.30 ± 0.67	4.77 ± 0.13	Zhangjiakou, Hebei, China
8	zyp2 gp 78-71	5.70 ± 0.04	4.35 ± 0.01	Zhangjiakou, Hebei, China
9	zyp2 gp 74-67	5.42 ± 0.04	4.56 ± 0.03	Zhangjiakou, Hebei, China
10	zyp2 gp 015-13	5.66 ± 0.42	4.59 ± 0.05	Zhangjiakou, Hebei, China
11	zyp2 gp 107-100	5.32 ± 0.62	4.01 ± 0.11	Zhangjiakou, Hebei, China
12	zyp2 gp 027-24	5.33 ± 0.48	4.33 ± 0.03	Zhangjiakou, Hebei, China
13	zyp2 gp 137-07	5.67 ± 0.56	3.82 ± 0.03	Zhangjiakou, Hebei, China
14	zyp2 gp 70-63	5.19 ± 0.16	4.68 ± 0.04	Zhangjiakou, Hebei, China
15	zyp2 gp 76-69	5.18 ± 0.08	3.59 ± 0.06	Zhangjiakou, Hebei, China
16	zyp2 gp 017-15	5.48 ± 0.50	4.61 ± 0.14	Zhangjiakou, Hebei, China
17	zyp2 gp 61-54	5.64 ± 0.09	4.61 ± 0.12	Zhangjiakou, Hebei, China
18	zyp2 gp 031-27	5.47 ± 0.52	4.71 ± 0.00	Zhangjiakou, Hebei, China
19	zyp2 gp 104-97	5.20 ± 0.37	4.4 ± 0.04	Zhangjiakou, Hebei, China
20	zyp2 gp 91-84	5.50 ± 0.57	4.51 ± 0.03	Zhangjiakou, Hebei, China
21	zyp2 gp 82-75	6.14 ± 0.26	4.34 ± 0.00	Zhangjiakou, Hebei, China
22	zyp2 gp 63-56	5.70 ± 0.57	5.25 ± 0.03	Zhangjiakou, Hebei, China
23	zyp2 gp 57-51	6.43 ± 0.43	4.87 ± 0.05	Zhangjiakou, Hebei, China
24	zyp2 gp 86-79	5.04 ± 0.45	4.94 ± 0.08	Zhangjiakou, Hebei, China
25	zyp2 gp 20B PSX 17G-26	5.42 ± 0.42	3.92 ± 0.04	Zhangjiakou, Hebei, China
26	zyp2 gp 083-2	6.18 ± 0.39	3.97 ± 0.14	Zhangjiakou, Hebei, China
27	zyp2 gp 024-21	5.84 ± 0.40	3.55 ± 0.02	Zhangjiakou, Hebei, China
28	IYP gp-6	5.58 ± 0.57	4.4 ± 0.02	Zhangjiakou, Hebei, China
29	zyp2 gp 67-60	5.69 ± 0.21	4.02 ± 0.11	Zhangjiakou, Hebei, China
30	Zhangyan #2	5.76 ± 0.12	4.1 ± 0.08	Zhangjiakou, Hebei, China
31	Yibaiyan #1	5.65 ± 0.47	4.49 ± 0.04	United States
32	Qinghai 444	5.62 ± 0.06	4.32 ± 0.01	Xining, Qinghai, China
33	Qingyin #1	5.45 ± 0.08	3.98 ± 0.08	Xining, Qinghai, China
34	Qingyin #1	5.51 ± 0.32	3.6 ± 0.00	Xining, Qinghai, China
35	Kanyan #1	5.69 ± 0.40	3.8 ± 0.04	
36	Yizhangyan #4	5.31 ± 0.19	3.64 ± 0.02	Zhangjiakou, Hebei, China
37	Dingyan #2	5.60 ± 0.01	4.32 ± 0.00	Dingxi, Gansu, China
38	Jingbaiyan #2	5.55 ± 0.06	3.69 ± 0.10	
39	Anrui	5.7 ± 0.13	4.28 ± 0.07	United States
40	Qinghai sweet oat	5.61 ± 0.08	4.12 ± 0.03	Xining, Qinghai, China
41	Kanyan #5	6.19 ± 0.52	3.78 ± 0.13	
42	Zhangyan #7	5.67 ± 0.08	3.1 ± 0.06	Zhangjiakou, Hebei, China
43	Qingyan #1	5.50 ± 0.11	4.69 ± 0.00	Xining, Qinghai, China
44	Baiyan #7	5.52 ± 0.18	4.38 ± 0.01	
45	Linna	5.57 ± 0.40	4.31 ± 0.04	Canada
46	Kanyan #4	5.47 ± 0.42	3.57 ± 0.03	
47	Baiyan #14	5.35 ± 0.42	3.48 ± 0.19	
48	Kanyan #2	5.19 ± 0.10	3.6 ± 0.08	
49	Zhangyan #3	5.21 ± 0.12	4.47 ± 0.06	Zhangjiakou, Hebei, China
50	Dingyin #1	5.15 ± 0.22	3.81 ± 0.02	Dingxi, Gansu, China
51	Kanyan #3	5.36 ± 0.08	4.13 ± 0.01	
52	Mingcui	5.60 ± 0.11	4.25 ± 0.10	United States
53	Yibaiyan #3	4.87 ± 0.59	4.67 ± 0.10	United States
54	Zhangyan #1	5.87 ± 0.52	4.03 ± 0.01	Zhangjiakou, Hebei, China
55	Kanyan #6	5.11 ± 0.40	4.08 ± 0.03	
56	Lezhen	5.53 ± 0.45	4.35 ± 0.00	United States
57	Zhangyan #4	5.96 ± 0.30	4.74 ± 0.01	Zhangjiakou, Hebei, China
58	Beile	5.50 ± 0.45	3.91 ± 0.00	Canada
59	Jiayan #2	5.37 ± 0.04	3.79 ± 0.11	Canada
60	ZNY 221	5.57 ± 0.06	3.97 ± 0.02	Zhangjiakou, Hebei, China
61	ZNY 202	6.17 ± 0.33	4.32 ± 0.04	Zhangjiakou, Hebei, China
62	ZNY 300	5.48 ± 0.46	4.14 ± 0.00	Zhangjiakou, Hebei, China
63	ZNY 303	5.61 ± 0.06	3.82 ± 0.04	Zhangjiakou, Hebei, China
64	ZNY 297	5.65 ± 0.43	4.48 ± 0.02	Zhangjiakou, Hebei, China
65	ZNY 218	5.54 ± 0.44	4.17 ± 0.00	Zhangjiakou, Hebei, China
66	ZNY 293	6.15 ± 0.47	4.78 ± 0.06	Zhangjiakou, Hebei, China
67	ZNY 225	5.57 ± 0.60	4.38 ± 0.00	Zhangjiakou, Hebei, China
68	ZNY 254	5.58 ± 0.66	3.27 ± 0.00	Zhangjiakou, Hebei, China
69	ZNY 233	5.19 ± 0.08	4.62 ± 0.04	Zhangjiakou, Hebei, China
70	ZNY 290	5.33 ± 0.23	3.7 ± 0.03	Zhangjiakou, Hebei, China
71	ZNY 205	5.78 ± 0.38	4.48 ± 0.17	Zhangjiakou, Hebei, China
72	ZNY 232	5.36 ± 0.28	4.13 ± 0.04	Zhangjiakou, Hebei, China
73	ZNY 248	5.40 ± 0.30	4.44 ± 0.07	Zhangjiakou, Hebei, China
74	ZNY 288	5.41 ± 0.34	4.23 ± 0.02	Zhangjiakou, Hebei, China
75	ZNY 231	5.44 ± 0.39	3.48 ± 0.10	Zhangjiakou, Hebei, China
76	ZNY 251	5.68 ± 0.72	4.51 ± 0.03	Zhangjiakou, Hebei, China
77	ZNY 258	5.62 ± 0.15	5.5 ± 0.08	Zhangjiakou, Hebei, China
78	ZNY 255	5.77 ± 0.60	4.83 ± 0.06	Zhangjiakou, Hebei, China
79	ZNY239	4.84 ± 0.31	4.01 ± 0.01	Zhangjiakou, Hebei, China

Data were expressed as mean ± standard deviation (*n* = 3), *w*/*w*; weight/weight.

**Table 2:** *β*-glucan content, moisture content, and origin of naked oat samples.

S. No.	Sample Name	Moisture Content (%)	*β*-Glucan % *w*/*w* (Dry wt. Basis)	Origin
1	Yanke #2	5.56 ± 0.31	3.96 ± 0.06	Huhehaote, Inner Mongolia, China
2	Baiyan #2	5.84 ± 0.39	4.00 ± 0.16	Baicheng, Jilin, China
3	Kanyou #6	5.74 ± 0.14	3.97 ± 0.09	
4	Dingyou #9	5.71 ± 0.29	3.59 ± 0.17	Dingxi, Gansu, China
5	Baiyan #11	5.63 ± 0.49	4.23 ± 0.03	Baicheng, Jilin, China
6	Kanyou #18	5.76 ± 0.05	5.06 ± 0.13	
7	Jinyan #8	5.97 ± 0.23	4.14 ± 0.10	Datong, Shanxi, China
8	Yuanza #1	5.14 ± 0.44	3.74 ± 0.05	Zhangjiakou, Hebei, China
9	Caoyou #1	5.91 ± 0.66	3.75 ± 0.11	Huhehaote, Inner Mongolia, China
10	Yanxuan 2007	5.61 ± 0.07	4.34 ± 0.01	Datong, Shanxi, China
11	Jinyan #1	5.64 ± 0.39	3.82 ± 0.03	Datong, Shanxi, China
12	Bayan #9	5.79 ± 0.13	5.21 ± 0.08	
13	Yizhangyou #6	5.60 ± 0.32	4.28 ± 0.08	Zhangjiakou, Hebei, China
14	Baiyan #15	5.55 ± 0.43	3.63 ± 0.05	
15	Baiyan #8	5.49 ± 0.68	3.82 ± 0.00	
16	Zhangyou #7	5.72 ± 0.66	3.93 ± 0.12	
17	Yizhangyou #3	5.82 ± 0.40	4.44 ± 0.01	Zhangjiakou, Hebei, China
18	Yizhangyou #12	5.52 ± 0.38	4.01 ± 0.04	
19	Yizhangyou #2	6.18 ± 0.57	3.84 ± 0.03	Zhangjiakou, Hebei, China
20	Jinyinyan #1	5.45 ± 0.39	4.54 ± 0.03	Datong, Shanxi, China
21	w85	5.44 ± 0.10	3.59 ± 0.01	Baicheng, Jilin, China
22	Yizhangyou #5	5.58 ± 0.42	4.25 ± 0.01	Zhangjiakou, Hebei, China
23	Kanyou #8	5.54 ± 0.43	4.23 ± 0.04	
24	Kanyou #13	5.66 ± 0.13	3.80 ± 0.02	
25	Huazao #2	5.51 ± 0.47	3.27 ± 0.01	Zhangjiakou, Hebei, China
26	Baiyan #13	5.77 ± 0.85	4.22 ± 0.01	
27	Kanyou #10	5.44 ± 0.54	4.14 ± 0.09	
28	Kanyou #5	5.22 ± 0.05	4.50 ± 0.02	
29	Neiyan #5	6.19 ± 0.42	3.98 ± 0.07	
30	Jinyan #14	5.33 ± 0.28	4.05 ± 0.10	Datong, Shanxi, China
31	Kanyou #3	5.43 ± 0.42	4.33 ± 0.04	
32	Ningyou #1	5.94 ± 0.61	4.18 ± 0.03	Guyuan, Ningxia, China
33	Yizhangyou #4	5.58 ± 0.07	3.73 ± 0.03	Zhangjiakou, Hebei, China
34	Yan 2009	5.39 ± 0.08	4.01 ± 0.01	Datong, Shanxi, China
35	Jinyan #13	5.13 ± 0.05	4.06 ± 0.06	Datong, Shanxi, China
36	Yizhangyou #5	5.31 ± 0.11	3.91 ± 0.03	Zhangjiakou, Hebei, China
37	Huawan #6	5.50 ± 0.56	3.78 ± 0.03	Zhangjiakou, Hebei, China
38	Yuanza #2	5.46 ± 0.04	4.43 ± 0.06	Zhangjiakou, Hebei, China
39	Ding you #1	5.46 ± 0.52	3.82 ± 0.00	Dingxi, Gansu, China
40	Jinyan #9	5.70 ± 0.87	3.13 ± 0.00	Datong, Shanxi, China
41	ZNY 242	5.83 ± 0.83	3.80 ± 0.06	Zhangjiakou, Hebei, China
42	ZNY 283	5.72 ± 0.72	3.55 ± 0.00	Zhangjiakou, Hebei, China
43	ZNY 273	5.17 ± 0.52	4.23 ± 0.04	Zhangjiakou, Hebei, China
44	ZNY 266	5.24 ± 0.08	4.31 ± 0.02	Zhangjiakou, Hebei, China
45	ZNY 272	5.81 ± 0.74	3.89 ± 0.02	Zhangjiakou, Hebei, China
46	ZNY 209	6.05 ± 0.57	3.84 ± 0.10	Zhangjiakou, Hebei, China
47	BY 1	5.59 ± 0.32	4.44 ± 0.09	Zhangjiakou, Hebei, China
48	BY 2	5.25 ± 0.39	4.98 ± 0.17	Zhangjiakou, Hebei, China
49	BY 3	5.37 ± 0.66	4.92 ± 0.07	Zhangjiakou, Hebei, China
50	BY 4	5.51 ± 0.49	5.22 ± 0.03	Zhangjiakou, Hebei, China
51	BY 5	5.32 ± 0.79	4.50 ± 0.04	Zhangjiakou, Hebei, China
52	BY 6	5.80 ± 0.49	4.45 ± 0.03	Zhangjiakou, Hebei, China
53	BY 7	5.75 ± 0.51	4.04 ± 0.10	Zhangjiakou, Hebei, China
54	BY 8	5.47 ± 0.49	4.28 ± 0.09	Zhangjiakou, Hebei, China
55	BY 9	6.03 ± 0.28	3.91 ± 0.11	Zhangjiakou, Hebei, China
56	BY 10	5.79 ± 0.42	4.31 ± 0.02	Zhangjiakou, Hebei, China
57	BY 11	5.62 ± 0.62	4.69 ± 0.05	Zhangjiakou, Hebei, China
58	BY 12	5.58 ± 0.58	4.19 ± 0.05	Zhangjiakou, Hebei, China
59	BY 13	5.49 ± 0.42	4.60 ± 0.09	Zhangjiakou, Hebei, China
60	BY 14	5.07 ± 0.19	4.52 ± 0.09	Zhangjiakou, Hebei, China
61	BY 15	5.54 ± 0.49	4.26 ± 0.04	Zhangjiakou, Hebei, China
62	BY 16	5.42 ± 0.79	4.47 ± 0.00	Zhangjiakou, Hebei, China
63	BY 17	5.13 ± 0.49	5.16 ± 0.00	Zhangjiakou, Hebei, China
64	BY 18	4.82 ± 0.51	4.44 ± 0.02	Zhangjiakou, Hebei, China
65	BY 19	5.27 ± 0.49	4.37 ± 0.03	Zhangjiakou, Hebei, China
66	BY 20	5.22 ± 0.28	4.03 ± 0.02	Zhangjiakou, Hebei, China
67	BY 21	5.55 ± 0.42	4.12 ± 0.01	Zhangjiakou, Hebei, China
68	BY 22	4.77 ± 0.62	4.07 ± 0.13	Zhangjiakou, Hebei, China
69	BY 23	5.31 ± 0.58	4.00 ± 0.05	Zhangjiakou, Hebei, China
70	BY 24	5.57 ± 0.42	4.13 ± 0.04	Zhangjiakou, Hebei, China
71	BY 25	4.80 ± 0.19	5.02 ± 0.12	Zhangjiakou, Hebei, China
72	BY 26	4.59 ± 0.25	4.61 ± 0.08	Zhangjiakou, Hebei, China
73	BY 27	5.76 ± 0.71	4.57 ± 0.06	Zhangjiakou, Hebei, China
74	BY 28	5.09 ± 0.55	4.37 ± 0.00	Zhangjiakou, Hebei, China
75	BY 29	5.00 ± 0.50	4.54 ± 0.00	Zhangjiakou, Hebei, China
76	BY 30	5.70 ± 0.47	3.43 ± 0.02	Zhangjiakou, Hebei, China
77	BY 31	5.41 ± 0.83	4.37 ± 0.02	Zhangjiakou, Hebei, China
78	BY 32	5.08 ± 1.07	4.04 ± 0.07	Zhangjiakou, Hebei, China
79	BY 33	4.43 ± 0.47	4.06 ± 0.12	Zhangjiakou, Hebei, China
80	BY 34	4.43 ± 0.27	5.17 ± 0.00	Zhangjiakou, Hebei, China
81	BY 35	5.01 ± 1.03	4.84 ± 0.00	Zhangjiakou, Hebei, China
82	BY 36	5.44 ± 0.10	4.37 ± 0.01	Zhangjiakou, Hebei, China
83	BY 37	5.26 ± 1.03	3.12 ± 0.00	Zhangjiakou, Hebei, China
84	BY 38	5.27 ± 0.39	3.13 ± 0.01	Zhangjiakou, Hebei, China
85	BY 39	4.69 ± 0.78	4.93 ± 0.01	Zhangjiakou, Hebei, China
86	BY 40	4.24 ± 0.37	3.90 ± 0.02	Zhangjiakou, Hebei, China
87	BY 41	4.85 ± 1.05	4.28 ± 0.02	Zhangjiakou, Hebei, China
88	BY 42	4.53 ± 1.31	4.45 ± 0.01	Zhangjiakou, Hebei, China
89	BY 43	4.54 ± 0.42	3.74 ± 0.04	Zhangjiakou, Hebei, China
90	BY 44	4.21 ± 0.11	4.03 ± 0.07	Zhangjiakou, Hebei, China
91	BY 45	5.01 ± 0.71	4.99 ± 0.08	Zhangjiakou, Hebei, China
92	BY 46	5.52 ± 0.66	4.16 ± 0.07	Zhangjiakou, Hebei, China
93	BY 47	5.64 ± 0.49	4.29 ± 0.06	Zhangjiakou, Hebei, China
94	BY 48	5.11 ± 0.42	4.25 ± 0.00	Zhangjiakou, Hebei, China
95	BY 49	4.22 ± 0.74	3.45 ± 0.04	Zhangjiakou, Hebei, China
96	BY 50	4.54 ± 0.16	3.73 ± 0.00	Zhangjiakou, Hebei, China
97	BY 51	4.92 ± 0.35	4.43 ± 0.00	Zhangjiakou, Hebei, China
98	BY 52	4.69 ± 0.79	4.13 ± 0.01	Zhangjiakou, Hebei, China
99	BY 53	5.02 ± 0.52	3.99 ± 0.02	Zhangjiakou, Hebei, China
100	ZNY282	4.17 ± 0.05	4.45 ± 0.05	Zhangjiakou, Hebei, China

Data were expressed as mean ± standard deviation (*n* = 3), *w*/*w*; weight/weight.

**Table 3:** Statistical results of partial least square regression models developed to predict the *β*-glucan content of hulled and naked oat samples.

Wavelength Range (nm)	Hulled Oats				Naked Oats			
	R^2^c	R^2^p	RMSEC	RMSEP	R^2^c	R^2^p	RMSEC	RMSEP
700–1300	0.789	0.735	0.177	0.199	0.677	0.620	0.210	0.228
1300–1900	0.431	0.301	0.291	0.323	0.325	0.226	0.304	0.325
1900–2500	0.460	0.336	0.284	0.315	0.382	0.274	0.291	0.315

## Data Availability

Data are available upon request.
